# Generating PET scan patterns in Alzheimer’s by a mathematical model

**DOI:** 10.1371/journal.pone.0299637

**Published:** 2024-04-16

**Authors:** Chaeyoung Lee, Avner Friedman

**Affiliations:** 1 Department of Mathematics, Kyonggi University, Suwon, Republic of Korea; 2 Department of Mathematics, The Ohio State University, Columbus, OH, United States of America; Georgetown University Medical Center, UNITED STATES

## Abstract

Alzheimer disease (AD) is the most common form of dementia. The cause of the disease is unknown, and it has no cure. Symptoms include cognitive decline, memory loss, and impairment of daily functioning. The pathological hallmarks of the disease are aggregation of plaques of amyloid-*β* (*Aβ*) and neurofibrillary tangles of tau proteins (*τ*), which can be detected in PET scans of the brain. The disease can remain asymptomatic for decades, while the densities of *Aβ* and *τ* continue to grow. Inflammation is considered an early event that drives the disease. In this paper, we develop a mathematical model that can produce simulated patterns of (*Aβ*,*τ*) seen in PET scans of AD patients. The model is based on the assumption that early inflammations, *R* and $\bar{R}$, drive the growth of *Aβ* and *τ*, respectively. Recently approved drugs can slow the progression of AD in patients, provided treatment begins early, before significant damage to the brain has occurred. In line with current longitudinal studies, we used the model to demonstrate how to assess the efficacy of such drugs when given years before the disease becomes symptomatic.

## Introduction

Alzheimer’s disease (AD) is the most common form of dementia. The pathological hallmarks of the disease are accumulation of amyloid-*β* (*Aβ*) plaques composed of *Aβ* peptides, and neurofibrillary tangles (NFT) composed of hyperphosphorylated tau proteins [1, 2]. The disease has no cure. An estimated 6.7 million Americans are living with Alzheimer’s in 2023 and, due to the rapid increase in aging population, this number is expected to reach 12.7 million by 2050 if no cure is found [3]. Until recently, all drugs to slow the progression of the disease failed in clinical trials. But in 2021, FDA approved a new drug, Aduhelm, that slows the progression of early stage of AD in some patients; Aduhelm is selectively binding amyloid aggregates in both oligomeric and fibrillatory states, rather than just in amyloid monomers [4].

PET scans of the brain of AD patients show patterns of accumulation of *Aβ* and NFT [5–8]; some patterns show high *Aβ* and low tau, while others show low *Aβ* and high tau. This gave rise to two different hypotheses. Based on PET scan patterns of high *Aβ* and low tau, the amyloid hypothesis states that *Aβ* aggregation triggers a chain of events that ultimately results in AD pathology, while based on patterns of low *Aβ* and high tau, the tau hypothesis postulates that tau tangle pathology precedes the *Aβ* plaques formation and that tau phosphorylation and aggregation are the main cause of AD. There is evidence for and against each of these hypotheses [9].

The earlier pathological signs of AD may appear 10 years, or more, before the onset of clinical symptoms [10, 11]. During this period, the hallmarks of the disease are the accumulation of *Aβ* and neurofibrillary tangles of tau proteins. The ability to identify early changes in the dynamics of *Aβ* and tau is an especially important goal for clinical trials [11]. Indeed, recent studies of longitudinal PET scans of *Aβ* and tau aim to determine whether patients with mild cognitive impairment (MCI) are AD positive [10, 12–16].

Cellular senescence, a state of permanent cell growth arrest, is associated with aging, and is believed to contribute to aging-related diseases, including AD [17]. Recent studies show that senescence in aged human neurons is a pathological feature of AD, and that these senescence neurons have a robust inflammatory response [18]; furthermore, targeting these deteriorating neurons could be an effective strategy for preventing AD [19].

Although the cause of AD is not known, we may assume that inflammation in neurons is an early event that drives the disease [20–23]. Introducing reactive oxygen species (ROS) as a potential cause of the disease, Hao et al. [24] developed a mathematical model of AD by a system of PDEs, that included, in addition to *Aβ* and NFT, the relevant brain cells, peripheral macrophages, and cytokines that activate, or are secreted by, these cells. They used the model to estimate the efficacy of experimental drugs, such as TNF-*α* inhibitor and TGF-*β* injection.

Thompson et al. [25] developed a mathematical model of protein-protein interactions between *Aβ* and NFT. The model consists of 4 equations for toxic and non-toxic *Aβ* and tau proteins; the parameters of the model are estimated from patterns seen in PET scans of AD patients. Bertsch et al. [26, 27] used the Smoluchowski equations with diffusion to simulate patterns of PET scans of *Aβ* seen in patients during progression of AD. In [28], they extended the model to study the synergy between *Aβ* and *τ* protein in AD patients. Stochastic approach to model AD was developed by Hadjichrysanthou et al. [29] to improve the design of clinical trials in Alzheimer’s. Article [29] includes a review of mathematical models of Alzheimer’s. In particular, [30] focuses on the role of prions in memory impairment; [31] studies how to fit neuropathological and epidemiological data in order to assess feasibility of intervention program; [32] finds that targeting microglia may hold promise in the prevention and treatment of AD; and [33] considers biomarkers to monitor *Aβ* burden in the brain, such as the levels of *Aβ* in CSF and plasma. A recent mathematical model proposes a convolutional neural network to identify Alzheimer’s—related mental disorder [34].

In this paper, we develop a mathematical model that can produce patterns of *Aβ* and *τ* as seen in PET scans of AD patients. We use the model to demonstrate that the abnormal aggregation of *Aβ* in AD patients can be either more significant, or less significant, than the abnormal aggregation of tau, in line with the amyloid hypothesis, or respectively, the tau hypothesis. We can also use the model in longitudinal studies, aimed at delaying the onset of AD pathology, by estimating the benefits of early treatment of AD, as soon as patients show signs of MCI, or earlier.

The mathematical model is a simplified version of [24], with more careful parameter estimates based on, and validated by, clinical data for *Aβ* [35], tau proteins [36], microglia [37], and neurons [38, 39]. More importantly, unlike [24], we assume that the early inflammation, which drives the disease, does not affect *Aβ* and tau in the same way; furthermore, we take the inflammations *R* associated with *Aβ*, and $\bar{R}$ associated with tau, as variable functions in space, that increase in time as follows:

$$\begin{array}{c} R\equiv R\left(t,x,y\right)=R\left(x,y\right)\frac{t/\gamma }{K+t},\;\bar{R}\equiv \bar{R}\left(t,x,y\right)=\bar{R}\left(x,y\right)\frac{t/\gamma }{K+t}, \end{array}$$

where *t* is time, (*x*, *y*) is a variable point in brain tissue (assumed, for simplicity, to be two-dimensional), and *K* and *γ* are positive constants.

The parameters *K*, *γ* determine the speed of the spread of inflammation and of the increase in PET scan values of (*Aβ*,*τ*) in AD patients. In most of the paper, we take *γ* = 1 and *K* = 100, but in the longitudinal simulations (Table 2 and Fig 9) of patients with long preclinical stages of up to 10 years, we slow the speed of the progression of the disease by taking *K* = 1000 and *γ* = 1/1.4.

We denote by $A_{\beta }^{i}$ and $A_{\beta }^{o}$ the concentrations of *Aβ* peptides inside and outside neurons, respectively, and by *τ* the concentration of tau protein in neurons. In health, $A_{\beta }^{i}$ and $A_{\beta }^{o}$ emerge from cleavage of neuronal membrane protein (APP) [40, 41]. We assume that AD develops from inflammation (ROS) in neurons, that causes abnormal increases in $A_{\beta }^{i}$ and *τ*. Hyperphosphorylated tau disrupts microtubule formation in neurons, forming instead neurofibrillary tangles, NFT (*F*_*i*_) [42, 43], which damage and cause death of neurons [44]. Extracellular $A_{\beta }^{o}$ detected in *Aβ*-plaques (e.g., *Aβ*-42) also induce death in neurons [45, 46].

Microglia (*M*) are attracted to $A_{\beta }^{o}$ and they are activated by $A_{\beta }^{o}$ [47] and by *F*_*i*_ [48]. Activated microglia clear $A_{\beta }^{o}$, but they also secrete IL-1*β*, TNF-*α* and other inflammatory cytokines that activate astrocytes (*A*), who then produce $A_{\beta }^{o}$ by cleaving their APP [49]. Another source of $A_{\beta }^{o}$ from neurons that die by necrosis [50, 51].

In addition to neurons, microglia and astrocytes, the other most common type of brain cells are oligodendrocytes, who secrete myelin that protects neurons. There is increasing evidence that myelin disruption is related to cognitive decline in AD. Oligodendrocyte progenitor disruption occurs in early stage in AD [52]. However, there is no evidence that oligodendrocytes are involved in *Aβ* plaques and NFT formations in AD.

**Fig 1:**
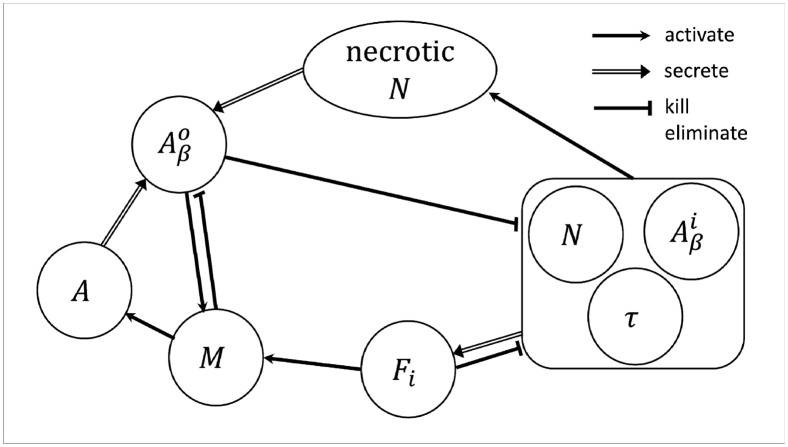
Network of variables in AD, composed of neurons (*N*), microglia (*M*), intraneural *Aβ* ($A_{\beta }^{i}$), extraneural *Aβ* ($A_{\beta }^{o}$), tau protein (*τ*), NFT (*F*_*i*_), and astrocytes (*A*).

Fig 1 is a schematic summary of the network associated with AD progression. The mathematical model is based on Fig 1.

## Mathematical model

Neurons and their extracellular spaces in the brain form a fine microscopic geometry, which makes it impossible to write and simulate equations. The same situation arises in electrophysiology, with myositis in cardiac tissue. In that case, the bidomain model has been introduced, and it is now considered to be the gold standard for numerical simulations [53]. We shall use the same concept in the present paper. Accordingly, each point in brain tissue is considered to be partially occupied by neurons, and partially extraneural. Thus, we view both the intraneural and extraneural spaces as spread smoothly over the same tissue. However, since neurons (*N*) are continuously dying, we shall have to take into account the fact that their share in each point in the brain tissue is decreased by the factor *N*/*N*_0_, where *N*_0_ is the density of *N* in health.

The model variables, in units of g/cm^3^, are:

*N*: Neurons,
*M:* Microglia (macrophages) in the extraneural space,
*A*: Astrocytes in the extraneural space,

$A_{\beta }^{i}$ : Amyloid-*β*, produced in *N*,

$A_{\beta }^{o}$ : Amyloid-*β* in the extraneural space,
*τ*: Tau proteins produced in *N*,
*F_i_*: Neurofibrillary protein (NFT) in *N*,

$\left(R,\bar{R}\right)$ : Reactive oxygen species (ROS) in *N*; *R* increases $A_{\beta }^{i}$ and $\bar{R}$ increases *τ*.

The vulnerability of the brain to ROS is a key factor and early event driving AD [20]. *Aβ* is constitutively produced in neurons upon cleavage of membrane soluble amyloid precursor protein (sAPP) into smaller fragments (peptides) and $A_{\beta }^{i}$ [40, 41]. We write the equation for $A_{\beta }^{i}$ in the following form:

$$\begin{array}{c} \frac{\partial A_{\beta }^{i}}{\partial t}-D_{A_{\beta }^{i}}\nabla^{2}A_{\beta }^{i}=[R+\lambda_{A_{\beta }^{i}}-d_{A_{\beta }^{i}}A_{\beta }^{i}]\frac{N}{N_{0}}, \end{array}$$ (1)

where *R* increases the proliferation of $A_{\beta }^{i}$; when *R* = 0, $\lambda_{A_{\beta }^{i}}-d_{A_{\beta }^{i}}{A_{\beta }^{i}}^{ss}=0,$ where ${A_{\beta }^{i}}^{ss}$ is the steady state of $A_{\beta }^{i}$ in health.

$A_{\beta }^{o}$ are the extraneural *Aβ* that form the plaque seen near neurons in AD patients. We write the equation for $A_{\beta }^{o}$ in the following form:

$$\begin{array}{cc} & \frac{\partial A_{\beta }^{o}}{\partial t}-D_{A_{\beta }^{o}}\nabla^{2}A_{\beta }^{o}=\lambda_{A_{\beta }^{o}}-\lambda_{A_{\beta }^{o}N}\frac{1}{N}\frac{\partial N}{\partial t}A_{\beta }^{i}-d_{A_{\beta }^{o}M}MA_{\beta }^{o}-d_{A_{\beta }^{o}}A_{\beta }^{o}+\lambda_{A_{\beta }^{o}A}AA_{\beta }^{o}, \end{array}$$ (2)

where ∂*N*/∂*t* is the death rate (−∂*N*/∂*t* > 0). Apoptotic cells are cleared by macrophages before their content leaks out [54], but some neurons die by necrosis [50], leaking their $A_{\beta }^{i}$ into the extraneural space [51], and since these $A_{\beta }^{i}$ are in the extracellular space, they increase the growth rate of $A_{\beta }^{o}$ in proportion to −∂*N*/∂*t*; they account for the second term on the right-hand side of Eq (2). The third term represents removal of $A_{\beta }^{o}$ by microglia [55–57]. The last term accounts for the secretion of $A_{\beta }^{o}$ by astrocytes [49].

Tau proteins are constitutively expressed in neurons [58]. Excessive $A_{\beta }^{i}$ activates GSK-3 through dephosphorylation, and activated GSK-3 promotes hyperphosphorylation of tau [59, 60], which results in formation of NFT [42, 43]. We can write the equation for tau as follows:

$$\begin{array}{c} \frac{\partial \tau }{\partial t}-D_{\tau }\nabla^{2}\tau =[\bar{R}+\lambda_{\tau }+\lambda_{\tau A_{\beta }^{i}}{\left(A_{\beta }^{i}-{A_{\beta }^{i}}^{ss}\right)}^{+}-d_{\tau }\tau ]\frac{N}{N_{0}}, \end{array}$$ (3)

where $\bar{R}$ increases the proliferataion of *τ*, and we use the notation: *X*^+^ = *X* *if* *X* ≥ 0, *X*^+^ = 0 *if* *X* < 0. Note that if $A_{\beta }^{i}={A_{\beta }^{i}}^{ss}$, then λ_*τ*_ − *d*_*τ*_*τ*^*ss*^ = 0, where *τ*^*ss*^ is the steady state of tau protein in health.

We assume, as in [24], that 60% of hyperphosphorylated tau proteins are involved in the formation of the neurofibrillary tangles, so that

$$\begin{array}{c} F_{i}\left(t\right)=0.6\left(\tau -\tau^{ss}\right). \end{array}$$ (4)

Microglia cells are highly dynamic both in normal and pathological brain conditions [61]. Microglia mobility in AD is directed toward a source of injury [62], which we take to be the accumulation of $A_{\beta }^{o}$. Microglia are activated by $A_{\beta }^{o}$ [47] and NFT [48]. Hence,

$$\begin{array}{cc} & \frac{\partial M}{\partial t}+\delta_{MA_{\beta }^{o}}\nabla \cdot (M\frac{\nabla A_{\beta }^{o}}{K_{\nabla A_{\beta }^{o}}+\left|\nabla A_{\beta }^{o}\right|})-D_{M}\nabla^{2}M\\ & \;=\lambda_{M}+M(\lambda_{MA_{\beta }^{o}}\frac{{\left(A_{\beta }^{o}-{A_{\beta }^{o}}^{ss}\right)}^{+}}{K_{A_{\beta }^{o}}+{\left(A_{\beta }^{o}-{A_{\beta }^{o}}^{ss}\right)}^{+}}+\lambda_{MF_{i}}\frac{F_{i}}{K_{F_{i}}+F_{i}})-d_{M}M. \end{array}$$ (5)

In AD, peripheral macrophages are known to migrate into the brain [54, 63]. For simplicity, we also include these peripheral macrophages in microglia.

Astrocytes are glial cells that support neurons in homeostasis. In AD, they are activated by inflammatory cytokines secreted by microglia, and activated astrocytes stimulate *Aβ* formation by cleaving their APP [49]. Other functions of subpopulations of activated astrocytes have been studied [49, 64, 65], but it is not clear, at this time, what is their total effect on AD progression. The effect of any inflammatory cytokine *I*, produced by *M*, on increased activation of *A* is proportional to $A\frac{I}{K_{I}+I}$, while

$$\begin{array}{c} \frac{dI}{dt}=\lambda_{I}M-d_{I}I. \end{array}$$

In steady state, we can represent the activation rate of *A* as being proportional to $A\frac{M}{K_{M}+M}$. We can then write the equation for *A* in the form:

$$\begin{array}{c} \frac{dA}{dt}=\lambda_{A}+\lambda_{AM}\frac{M}{K_{M}+M}A-d_{A}A. \end{array}$$ (6)

NFT causes death of neurons [44]. It is widely thought that amyloid plaques also contribute to the death of neurons in people with Alzheimer’s [66]. In particular, *Aβ* 42/40 are detected in Alzheimer’s amyloid plaques [46], and *Aβ*-42 induces apoptosis in neurons by targeting their mitochondria [45]. Furthermore, $A_{\beta }^{o}$ causes inflammation indirectly, for example, by activating microglia, who produce inflammatory cytokines [67, 68], which further damages neurons. We write the equation for *N* in the following form:

$$\begin{array}{c} \frac{\partial N}{\partial t}=-d_{NF_{i}}\frac{F_{i}}{K_{F_{i}}+F_{i}}N-d_{NA_{\beta }^{o}}\frac{{\left(A_{\beta }^{o}-{A_{\beta }^{o}}^{ss}\right)}^{+}}{K_{A_{\beta }^{o}}+{\left(A_{\beta }^{o}-{A_{\beta }^{o}}^{ss}\right)}^{+}}N,\;N\left(0\right)=N_{0}. \end{array}$$ (7)

The most common human brain cells are neurons and glial cells. There are approximately 100 billion neurons in adult humans, and at least as many glial cells [69]. Taking the mass one neuron to be $10^{-9}\;g$, and noting that the brain volume is 1,500$\;{\text{cm}}^{3}$, we find that the density of neurons in health is

$$\begin{array}{c} N_{0}=6.00\times 10^{-2}\;g/{\text{cm}}^{3}. \end{array}$$

Microglia make 6% of all brain cells [70], which we take to be 200 billion, and hence their density is approximately 12% of the density of neurons, so that

$$\begin{array}{c} M_{0}=7.20\times 10^{-3}\;g/{\text{cm}}^{3}. \end{array}$$

Astrocytes are four times as many as microglia [71]. Hence,

$$\begin{array}{c} A_{0}=2.88\times 10^{-2}\;g/{\text{cm}}^{3}. \end{array}$$

The number of neurons decreases by approximately 34% over the entire period of AD [39]. However, life expectancy at diagnosis varies greatly [38, 72, 73]. We take it to be in the range of 5–20 years, with an average of 10 years [38], so that

$$\begin{array}{c} N\left(t\right)=3.96\times 10^{-2}\;g/{\text{cm}}^{3}\;\;\text{at}\;\;t=10\;\;\text{years}. \end{array}$$ (8)

Assuming that this corresponds to the constant death rate *d*_*N*_, then, for life expectancy of 15 years, the death rate of *N* will be $\frac{10}{20}d_{N}$, resulting in *N*(*t*) = 4.87 × 10^−2^ at *t* = 10 years; and for life expectancy of 5 years, death rate will be $\frac{10}{5}d_{N}$, so that *N*(*t*) = 2.61 × 10^−2^ at *t* = 10 years. (Of course, the simulation in this case will stop after 5 years.) Hence,

$$\begin{array}{c} \text{Average}\;N\left(t\right)=3.96\times 10^{-2}\;g/{\text{cm}}^{3}\;\;(\text{range}\;2.61\times 10^{-2}-4.87\times 10^{-2})\;\text{in}\;\text{AD}. \end{array}$$ (9)

In AD, microglia show high proliferation and differentiation [57, 74]; we take the range of microglia density to be, approximately, 1.5–3 times the density in homeostasis. The concentration of the pro-inflammatory monocytes is approximately 2.2 larger in AD than in health [37]. Accordingly, we take

$$\begin{array}{c} \text{Average}\;M=15.84\times 10^{-3}\;g/{\text{cm}}^{3}\;\;(\text{range}\;9.8\times 10^{-3}-19.8\times 10^{-3})\;\text{in}\;\text{AD}. \end{array}$$ (10)

Concentration of *Aβ* in gray matter was reported in [35], in health and in AD, as follows:

$$\begin{array}{c} \begin{array}{cc} & A_{\beta }^{o}=1,000\;\text{ng}/g\;(50-3,500\;\text{range})\;\;\text{in}\;\text{health},\\ & A_{\beta }^{o}=6,700\;\text{ng}/g\;(1,100-23,000\;\text{range})\;\;\text{in}\;\text{AD}. \end{array} \end{array}$$ (11)

Concentration of tau protein was reported in [36], in health and in AD:

$$\begin{array}{c} \begin{array}{cc} & \tau =137\;\text{pg}/\text{ml}\;(50-300\;\text{range})\;\;\text{in}\;\text{health},\\ & \tau =490\;\text{pg}/\text{ml}\;(300-1,000\;\text{range})\;\;\text{in}\;\text{AD}. \end{array} \end{array}$$ (12)

## Results

### Numerical simulations

The parameters of the model are estimated in S1B File, their values are listed in S1 Table in S1 File (S1D File), and the numerical method is described in S1C File. We simulated the ODE version of the model for 3650 days (10 years) in order to show agreement with the data in Eqs (9)–(12). We assume that inflammation begins at day 0 and increases with time, taking

$$\begin{array}{c} R\left(t\right)=R\frac{t}{100+t},\;\bar{R}\left(t\right)=\bar{R}\frac{t}{100+t}; \end{array}$$ (13)

in health both *R* and $\bar{R}$ are equal to 0.

We found that with $R=R^{*}=1.85\times 10^{-6}\;g/({\text{cm}}^{3}\cdot d)$ and $\bar{R}={\bar{R}}^{*}=4.13\times 10^{-10}\;g/({\text{cm}}^{3}\cdot d)$, we get an agreement with the average clinical data, as shown in Table 1. We also found that if we modify (*R*, $\bar{R}$) in a certain region, we still get agreement with the range of data in Eqs (9)–(12). In particular, as seen in Table 1, the cases of $R=1.5R^{*},\;\bar{R}=0.5{\bar{R}}^{*}$ (high *Aβ*, low *τ*) and $R=0.5R^{*},\;\bar{R}=1.5{\bar{R}}^{*}$ (low *Aβ*, high *τ*) are included in the physiological range of inflammation in AD patients.

**Table 1:** Results of ODE system in units of $g/{\text{cm}}^{3}$.

Variables	Average clinical data		Numerical results at t=3650d			
	In health	In AD	*R* = 0	R* = 1.85 × 10^−6^	*R* = 1.5R*	*R* = 0.5R*
			$\bar{R}=0$	${\bar{R}}^{*}=4.13\times 10^{-10}$	$\bar{R}=0.5{\bar{R}}^{*}$	$\bar{R}=1.5{\bar{R}}^{*}$
$A_{\beta }^{i}$	1.00 × 10^−6^		1.00 × 10^−6^	1.97 × 10^−6^	2.46 × 10^−6^	1.49 × 10^−6^
$A_{\beta }^{o}$	1.00 × 10^−6^	6.70 × 10^−6^	1.00 × 10^−6^	6.64 × 10^−6^	7.90 × 10^−6^	5.19 × 10^−6^
*τ*	1.37 × 10^−10^	4.90 × 10^−10^	1.37 × 10^−10^	4.81 × 10^−10^	3.63 × 10^−10^	5.98 × 10^−10^
*F*_*i*_	0		0	2.06 × 10^−10^	1.36 × 10^−10^	2.77 × 10^−10^
*M*	7.20 × 10^−3^	15.84 × 10^−3^	7.20 × 10^−3^	15.66 × 10^−3^	14.77 × 10^−3^	15.63 × 10^−3^
*A*	2.88 × 10^−2^		2.88 × 10^−2^	3.99 × 10^−2^	3.87 × 10^−2^	3.98 × 10^−2^
*N*	6.00 × 10^−2^	3.96 × 10^−2^	6.00 × 10^−2^	4.01 × 10^−2^	4.10 × 10^−2^	4.00 × 10^−2^

**Fig 2:**
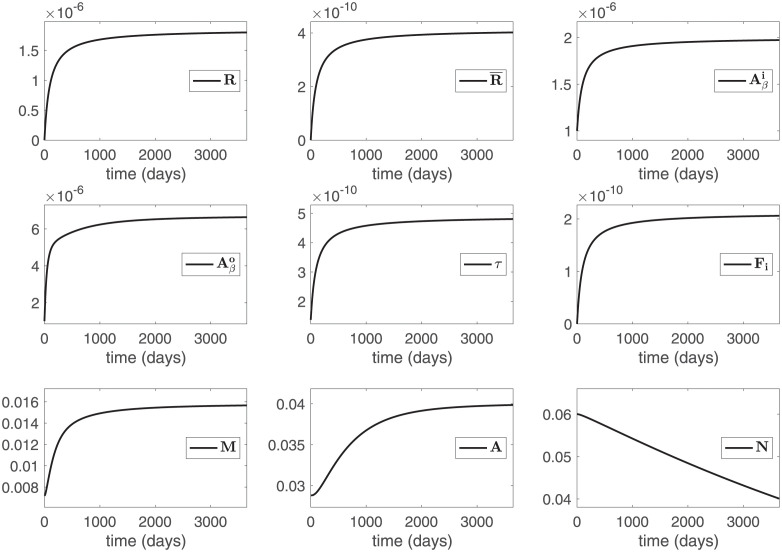
Profiles of the model variables with the ODE model and its parameters from S1D File: *R*(*t*) = *Rt*/(100 + *t*) and $\bar{R}\left(t\right)=\bar{R}t/\left(100+t\right)$ for $R=1.85\times 10^{-6}\;g/({\text{cm}}^{3}\cdot d)$ and $\bar{R}=4.13\times 10^{-10}\;g/({\text{cm}}^{3}\cdot d)$.

Fig 2 shows the profiles of the model variables with *R* = R* and $\bar{R}={\bar{R}}^{*}$ for 10 years. We see that $A_{\beta }^{o}$, *τ*, and *M* sharply increase in the first 200–300 days, and then continue to slowly increase, reaching the average clinical values $A_{\beta }^{o}=6.70\times 10^{-6}\;g/{\text{cm}}^{3}$, $\tau =4.90\times 10^{-10}\;g/{\text{cm}}^{3}$, and $M=15.84\times 10^{-3}\;g/{\text{cm}}^{3}$. Neuronal population, *N*, is decreasing from $6.00\times 10^{-2}\;g/{\text{cm}}^{3}$ to $4.01\times 10^{-2}\;g/{\text{cm}}^{3}$ after 10 years, in close agreement with the average value in Eq (9). Astrocytes, *A*, activated by microglia, are increasing more slowly toward a steady state.

We note that the computational results in Table 1 remain the same if we replace 100 days in Eq (13) by a larger number of days (e.g., 500, 1000); the only difference in the profile of the variables will be in the earlier days of the disease.

We next proceed with PDE simulations in a two-dimensional domain *Ω* using the no-flux boundary conditions,

$$\begin{array}{c} \frac{\partial A_{\beta }^{i}}{\partial n}=0,\;\frac{\partial A_{\beta }^{o}}{\partial n}=0,\;\frac{\partial \tau }{\partial n}=0,\;\frac{\partial M}{\partial n}=0, \end{array}$$ (14)

where n is the outer normal vector at the boundary ∂*Ω*, initial conditions in health, and inflammation in the form

$$\begin{array}{c} R\left(x,y,t\right)=R\left(x,y\right)\frac{t}{100+t}\;\text{and}\;\bar{R}\left(x,y,t\right)=\bar{R}\left(x,y\right)\frac{t}{100+t}. \end{array}$$

We take different patterns of *R*(*x*, *y*) and $\bar{R}\left(x,y\right)$, that include in regions with high *Aβ* and low *τ*, and regions with low *Aβ* and high *τ*.

An inflammation map consists of a square divided into pixels. Each pixel is given one of the following four choices:

(i) *Aβ*-biased pixel: *R*(*x*, *y*) = 1.5R* and $\bar{R}\left(x,y\right)=0.5{\bar{R}}^{*}$ with symbol ○,
(ii) *τ*-biased pixel: *R*(*x*, *y*) = 0.5R* and $\bar{R}\left(x,y\right)=1.5{\bar{R}}^{*}$ with symbol ●,
(iii) non-biased pixel: *R*(*x*, *y*) = R* and $\bar{R}\left(x,y\right)={\bar{R}}^{*}$ with symbol ✫,
(iv) zero-inflammation pixel: *R*(*x*, *y*) = 0 and $\bar{R}\left(x,y\right)=0$ with symbol ✕.

**Fig 3:**
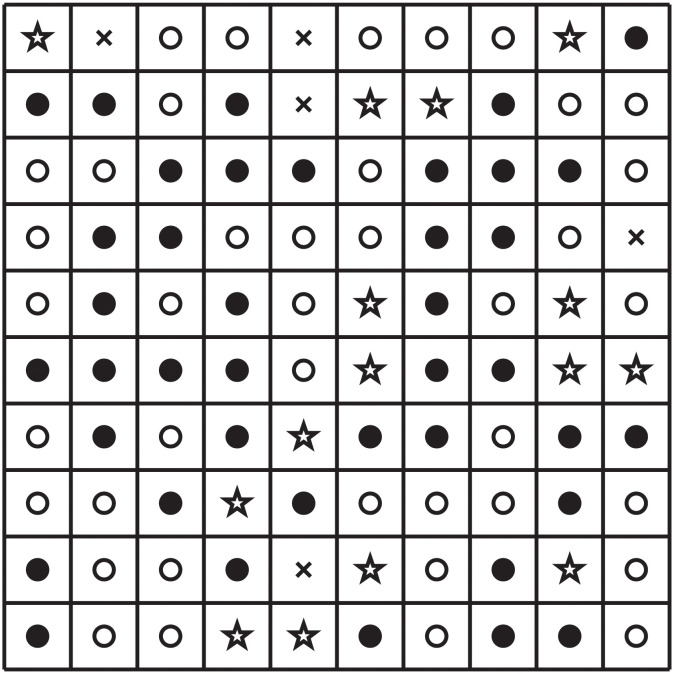
Inflammation map for *R* and $\bar{R}$ with 40% *Aβ*-biased, 40% *τ*-biased, 15% non-biased, and 5% zero-inflammation pixels.

We say that an inflammation map is *Aβ*-biased if the percentage, of pixels with *Aβ*-bias is larger than the percentage of pixels with *τ*-bias; it is *τ*-biased if the percentage of pixels with *τ*-bias is larger than the percentage of pixels with *Aβ*-bias; and it is non-biased if both percentages are equal. We use the same definition also when the numbers 1.5 and 0.5 are replaced by *N*_1_ and *N*_2_ where *N*_1_ > 1 > *N*_2_. Figs 3, 5A and 6A display, respectively, three different inflammation maps: non-biased, *Aβ*-biased, and *τ*-biased.

**Fig 4:**
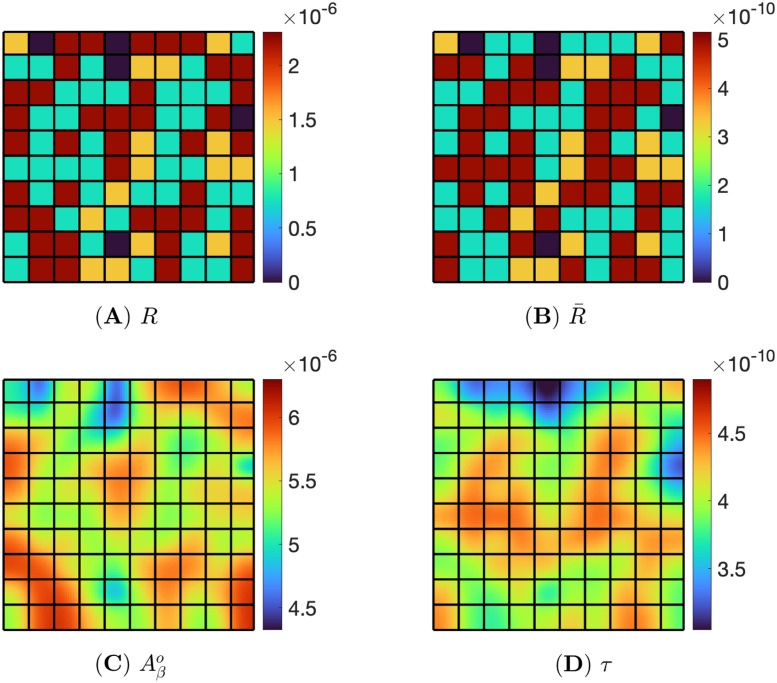
Non-biased inflammation. Pixels consist of 40% *Aβ*-bias, 40% *τ*-bias, 15% non-bias, and 5% zero inflammation. Snapshots are (A) *R*, (B) $\bar{R}$, (C) $A_{\beta }^{o}$, and (D) *τ* at t=500d.

Fig 3 consists of 40% *Aβ*-biased, 40% *τ*-biased, 15% non-biased, and 5% zero-inflammation pixels, which are randomly chosen. We solved the PDE system (1)–(7) with the boundary conditions (14) by the numerical method (S1C File) using the parameters from S1D File, with this inflammation map, and then the computational results at t=500d are shown in Fig 4.

Fig 4A and 4B show the non-biased inflammation of Fig 3 in color. In Fig 4A, for inflammation *R*, we denote each pixel by a color: red for *Aβ*-bias, blue for *τ*-bias, yellow for non-bias, and navy for health (no inflammation). Fig 4B shows $\bar{R}$ in a color map (with the same colors). Fig 4C and 4D are the snapshots of evolution of $A_{\beta }^{o}$ and *τ* corresponding to *R* and $\bar{R}$ at time t=500d. Here, the range of $A_{\beta }^{o}$ is between 4.64 × 10^−6^ and 6.20 × 10^−6^, and that of *τ* is 3.05 × 10^−10^ and 4.62 × 10^−10^. The non-biased inflammation, where the ratio of *R* and $\bar{R}$ is the same, induces the similar-sized red area in both $A_{\beta }^{o}$ and *τ*, as shown in Fig 4C and 4D.

**Fig 5:**
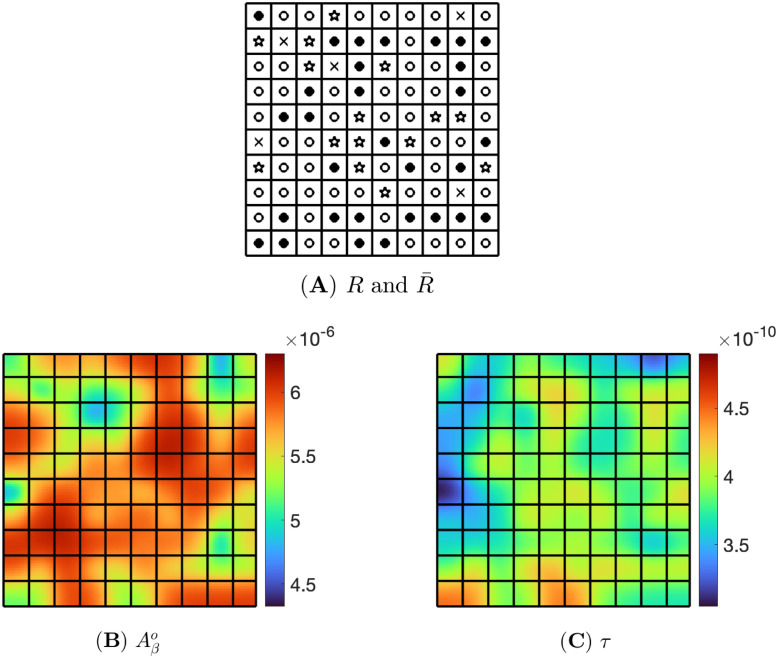
*Aβ*-biased inflammation. Pixels in (A) consist of 50% *Aβ*-bias, 30% *τ*-bias, 15% non-bias, and 5% zero inflammation, (B) and (C) are snapshots of $A_{\beta }^{o}$ and *τ* at t=500d, respectively.

Next, Fig 5A illustrates the *Aβ*-biased inflammation that consists of 50% *Aβ*-biased, 30% *τ*-biased, 15% non-biased, and 5% zero-inflammation pixels. At time t=500d, the density of $A_{\beta }^{o}$ is between 4.92 × 10^−6^ and 6.27 × 10^−6^, and the density of *τ* is between 3.16 × 10^−10^ and 4.61 × 10^−10^. Since *Aβ*-biased pixels outnumber *τ*-biased pixels, the density of $A_{\beta }^{o}$ is high across the domain, and most of the pixels are red as seen in Fig 5B. On the other hand, the *τ* densities on most pixels of the domain are distributed in the middle of the range as shown in Fig 5C.

**Fig 6:**
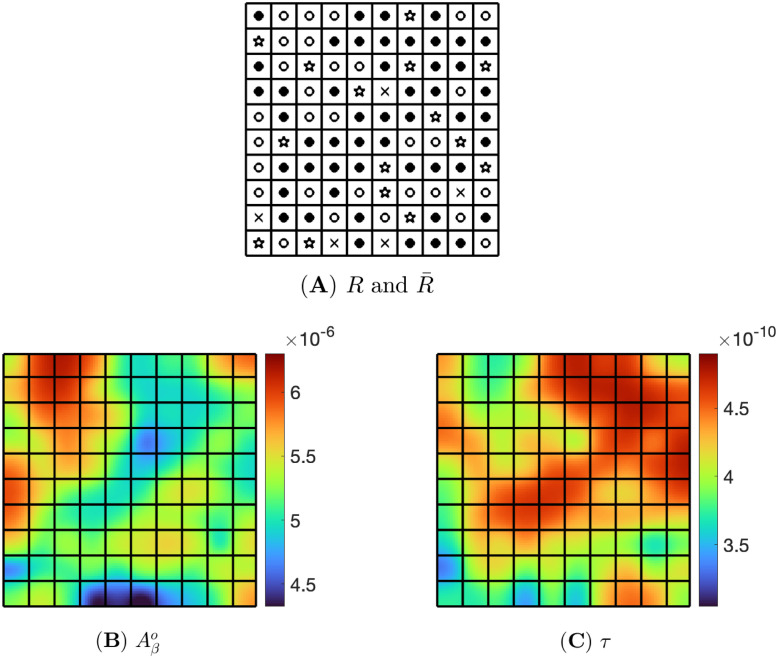
*τ*-biased inflammation. Pixels in (A) consist of 30% *Aβ*-bias, 50% *τ*-bias, 15% non-bias, and 5% zero inflammation. (B) and (C) are snapshots of $A_{\beta }^{o}$ and *τ* at t=500d, respectively.

Fig 6A illustrates, by contrast, a case of *τ*-biased inflammation where 50% pixels are *τ*-biased, 30% pixels are *Aβ*-biased, and the rest are the same as above. At time t=500d, the density of $A_{\beta }^{o}$ is between 4.33 × 10^−6^ and 6.30 × 10^−6^, and the density of *τ* is between 3.41 × 10^−10^ and 4.90 × 10^−10^. Compared to the *Aβ*-biased inflammation in Fig 5B, the range of $A_{\beta }^{o}$ densities looks wider, however, this is caused by only a few pixels, and in fact, relatively low densities can be seen in most pixels of Fig 6B. The density of *τ*, as shown in Fig 6C, is higher than in Figs 4 and 5.

**Fig 7:**
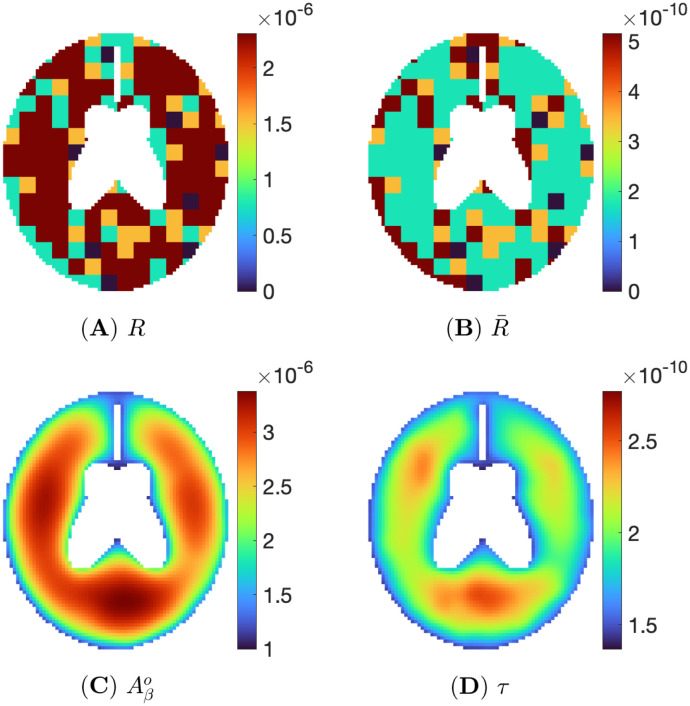
With $A_{\beta }^{o}$-biased inflammation, snapshots for (A) *R*, (B) $\bar{R}$, (C) $A_{\beta }^{o}$, and (D) *τ* at t=500d.

**Fig 8:**
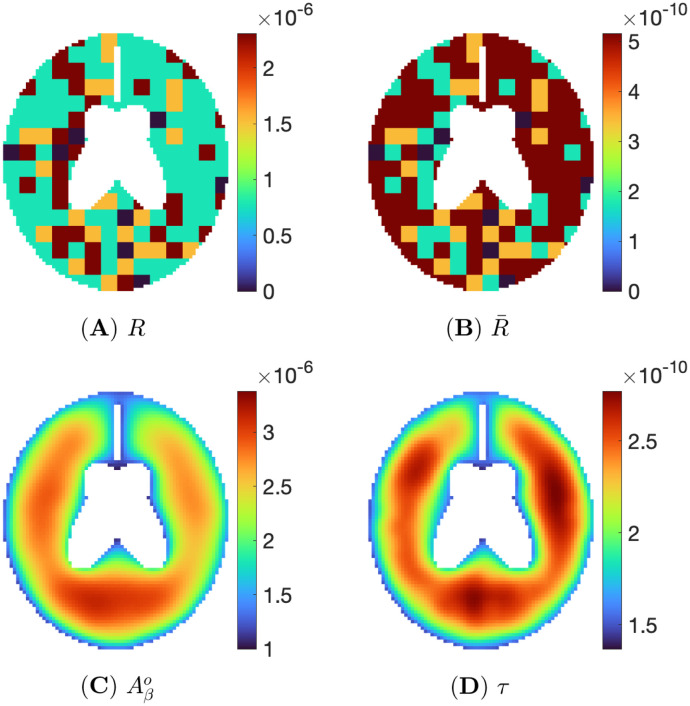
With *τ*-biased inflammation, snapshots for (A) *R*, (B) $\bar{R}$, (C) $A_{\beta }^{o}$, and (D) *τ* at t=500d.

Brain PET scans can indicate the distribution and density of $A_{\beta }^{o}$ and *τ* in the AD patients. This graphical analysis, along with cognitive and motion tests, are used in AD diagnosis. Various patterns of PET scans of AD patients are given in [75]. We can use our model to produce similar patterns by choosing appropriate inflammation functions. For instance, Figs 7 and 8 show two simple cases corresponding to (high $A_{\beta }^{o}$, low *τ*) and (low $A_{\beta }^{o}$, high *τ*). As shown in Fig 7A and 7B, inside the brain area, we allocate 60% of (high *R*, low $\bar{R}$), 20% of (low *R*, high $\bar{R}$), 15% non-biased inflammation with orange color, and 5% zero inflammation with green color in order to generate the situation of the A*β*-hypothesis. For *R* and $\bar{R}$, high density is colored with red, and low density is with yellow color. We solve the PDE system, and after 500 days, the density distribution of $A_{\beta }^{o}$ and of *τ* inside the brain take the form seen in Fig 7C and 7D. In line with the *Aβ*-hypothesis, the density of $A_{\beta }^{o}$ is high throughout the domain, where it is displayed in red, while in the distribution of *τ* density, some areas with high density are red, and other areas with low density are displayed in yellow and green.

Fig 8 is a computational simulation in line with the *τ*-hypothesis. We assign, in Fig 8A and 8B, 30% of (high *R*, low $\bar{R}$), 60% of (low *R*, high $\bar{R}$), 15% non-biased inflammation, and 5% zero inflammation inside the brain. Fig 7C and 7D show the density distribution of $A_{\beta }^{o}$ and *τ* inside the brain at time *t* = 500 day. The density of *τ* is very large throughout the domain. The density of $A_{\beta }^{o}$ is rather high in some areas but low in others, and altogether it is less than in the case of *Aβ*-biased inflammation of Fig 7.

### Longitudinal simulation

Drugs that slow the progression of AD could be useful in monitoring disease evolution [10]. Ongoing trials are looking at whether treating people with preclinical Alzheimer’s may delay or slow the onset of symptoms [10, 11]. Such trials can trace the dynamics of *Aβ* and tau by PET scans, and there is a need for longitudinal amyloid and tau PET imaging that can correlate to temporal changes in the brain, e.g., cognitive and movement changes [12].

We proceed to illustrate how the early stage of AD can lead, in 10 years of PET scan tests taken every 100 days, to levels of *Aβ* and tau significantly higher than the average levels of clinical AD. We take a slowing progressive inflammation of the following form:

$$\begin{array}{c} R\left(x,y\right)\frac{t/\gamma }{1000+t},\;\;\text{and}\;\;\bar{R}\left(x,y\right)\frac{t/\gamma }{1000+t}, \end{array}$$

where *R*(*x*, *y*) and $\bar{R}\left(x,y\right)$ are taken as in Fig 3 (non-biased inflammation), and *γ* is a positive parameter; we take *γ* = 1/1.4 in this example. Table 2 lists the average density of $A_{\beta }^{o}$ and *τ* each 100 days.

**Table 2:** Average densities of $A_{\beta }^{o}$ and *τ* over time when non-biased inflammation is given.

Time	100 d	200 d	300 d	400 d	500 d	600 d	700 d	800 d	900 d
$A_{\beta }^{o}$	2.15e-06	2.83e-06	3.33e-06	3.73e-06	4.06e-06	4.35e-06	4.59e-06	4.80e-06	4.99e-06
*τ*	1.79e-10	2.15e-10	2.45e-10	2.71e-10	2.93e-10	3.13e-10	3.30e-10	3.46e-10	3.59e-10
Time	1000 d	1100 d	1200 d	1300 d	1400 d	1500 d	1600 d	1700 d	1800 d
$A_{\beta }^{o}$	5.16e-06	5.30e-06	5.44e-06	5.56e-06	5.67e-06	5.77e-06	5.87e-06	5.95e-06	6.03e-06
*τ*	3.72e-10	3.83e-10	3.93e-10	4.02e-10	4.11e-10	4.19e-10	4.26e-10	4.33e-10	4.39e-10
Time	1900 d	2000 d	2100 d	2200 d	2300 d	2400 d	2500 d	2600 d	2700 d
$A_{\beta }^{o}$	6.10e-06	6.17e-06	6.23e-06	6.29e-06	6.35e-06	6.40e-06	6.45e-06	6.49e-06	6.53e-06
*τ*	4.45e-10	4.50e-10	4.55e-10	4.60e-10	4.64e-10	4.68e-10	4.72e-10	4.76e-10	4.80e-10
Time	2800 d	2900 d	3000 d	3100 d	3200 d	3300 d	3400 d	3500 d	3600 d
$A_{\beta }^{o}$	6.57e-06	6.61e-06	6.65e-06	6.68e-06	6.71e-06	6.74e-06	6.77e-06	6.80e-06	6.83e-06
*τ*	4.83e-10	4.86e-10	4.89e-10	4.92e-10	4.95e-10	4.97e-10	5.00e-10	5.02e-10	5.04e-10

**Fig 9:**
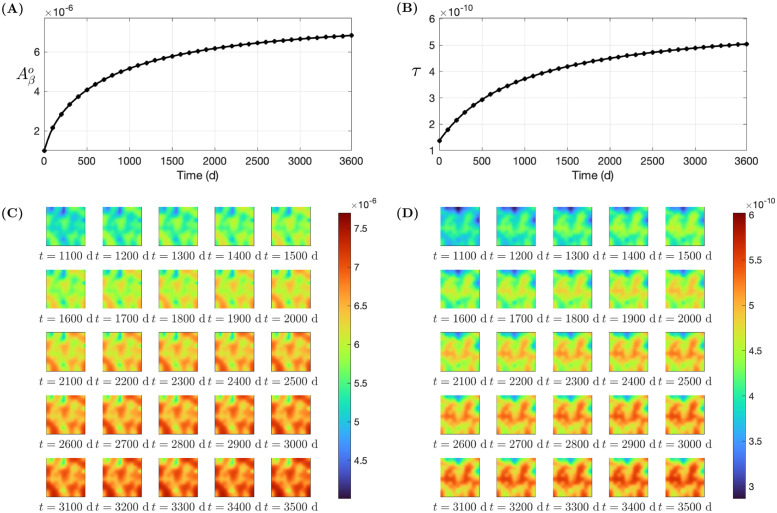
Longitudinal simulation with non-biased inflammation from *t* = 0 to *t* = 3600: (A)–(B) average densities and (C)–(D) color maps of $A_{\beta }^{o}$ and *τ*, respectively.

The dynamics of these averages are seen in Fig 9A and 9B, and the pointwise densities of $A_{\beta }^{o}$ and *τ* in the square domain are shown in Fig 9C and 9D. From Fig 9A and 9B, we see that $A_{\beta }^{o}$ and *τ* averages increase fast in the first few years, and slower in later years. There is no precise relationship between the level of AD progression and the level of ($A_{\beta }^{o},\tau$). From Table 2, we see that the value of ($A_{\beta }^{o},\tau$) after 10 years is well within the range of values for symptomatic AD patients, as documented in Eqs. (11) and (12). We accordingly make the assumption that after 10 years the disease has become pathological, while MCI symptoms may have developed already a few years earlier. Fig 9 describes the progression of AD over a square; similarly we can simulate the progression of AD in other regions of the brain, or the whole brain, as in Figs 7 and 8. We can also produce similar figures in the case of *Aβ*-biased and *τ*-biased inflammation maps.

New AD drugs (Aducanumab, Leqembi [76]) are much more effective if treatment begins before damage to the brain had already occurred. The drugs do not cure the disease (they do not eliminate the inflammation), but they slow the aggregation of $A_{\beta }^{o}$ and *τ*, and the progression of the disease.

Longitudinal studies are concerned with the dilemma of early treatment of AD patients, before it becomes pathological. Consider, for example, the case of a patient with MCI symptoms. It is not clear whether this is a case of AD or another form of dementia. In the first case, starting treatment with AD drug would be beneficial, but, in the second case, the drug has no benefits and may even be harmful because of negative side effects. Longitudinal studies aim to address this dilemma. Here, we use our model to quantify the benefits in early treatment in terms of extending the period of time that an AD patient remains asymptomatic.

**Fig 10:**
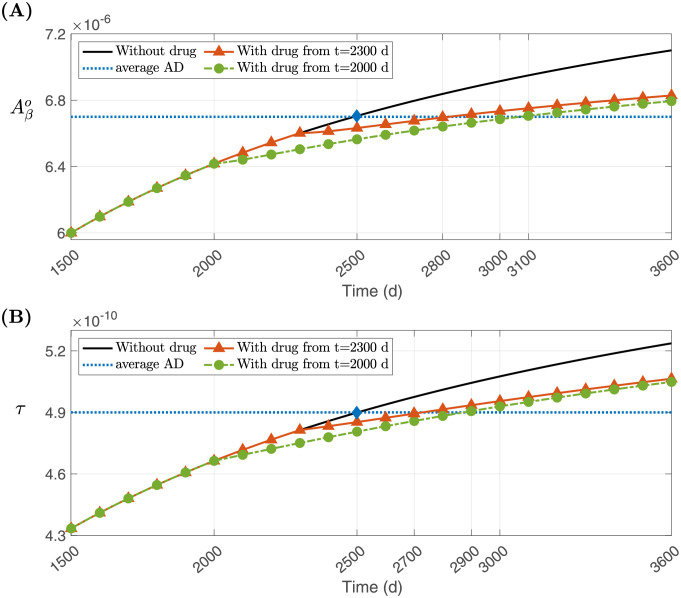
Comparison between densities of (A) $A_{\beta }^{o}$ and (B) *τ* over time with and without the AD drug treatment. There are two cases of treatments depending on starting time: For treatment starting on t=2000d (green bullets), $A_{\beta }^{o}$ reaches average AD at t=3100d and *τ* is t=2900d; for treatment starting later, at t=2300d (orange solid triangles), $A_{\beta }^{o}$ reaches average AD at t=2800d and *τ* is t=2700d.

We consider a drug *D* whose effect is to reduce $A_{\beta }^{o}$ and *τ*, and accordingly modify Eqs (2)–(3) during treatment that begins at time *t* = *t*_0_, for instance, as follows:

$$\begin{array}{cc} \frac{\partial A_{\beta }^{o}}{\partial t}-D_{A_{\beta }^{o}}\nabla^{2}A_{\beta }^{o} & =\lambda_{A_{\beta }^{o}}-\lambda_{A_{\beta }^{o}N}\frac{1}{N}\frac{\partial N}{\partial t}(\frac{1}{1+D^{*}\left(t-t_{0}\right)/\left(K_{D}+\left(t-t_{0}\right)\right)}A_{\beta }^{i})\\ & \;-d_{A_{\beta }^{o}M}MA_{\beta }^{o}-d_{A_{\beta }^{o}}A_{\beta }^{o}\\ & \;+\lambda_{A_{\beta }^{o}A}A(\frac{1}{1+\mu D^{*}\left(t-t_{0}\right)/\left(K_{D}+\left(t-t_{0}\right)\right)}A_{\beta }^{o}), \end{array}$$ (15)

$$\begin{array}{cc} \frac{\partial \tau }{\partial t}-D_{\tau }\nabla^{2}\tau & =[\bar{R}+\lambda_{\tau }+\frac{\lambda_{\tau A_{\beta }^{i}}{\left(A_{\beta }^{i}-{A_{\beta }^{i}}^{ss}\right)}^{+}}{1+\mu^{*}D^{*}\left(t-t_{0}\right)/\left(K_{D}+\left(t-t_{0}\right)\right)}-d_{\tau }\tau ]\frac{N}{N_{0}}, \end{array}$$ (16)

for some parameter *μ* and μ*, and dose D* of the drug. For a conceptual example, we simply use the ODE system with the non-biased inflammations

$$\begin{array}{c} R\left(t\right)=R^{*}\frac{t/\gamma }{1000+t}\;\;\text{and}\;\;\bar{R}\left(t\right)={\bar{R}}^{*}\frac{t/\gamma }{1000+t}, \end{array}$$

where 1/*γ* = 1.4, and the initial conditions where all variables are in health, as listed in Table 1. Fig 10 shows the profiles of $A_{\beta }^{o}$ and *τ* densities with no drug, corresponding to Table 2 and Fig 9A and 9B (black solid lines), and the average densities in AD (blue dotted horizontal lines). The $A_{\beta }^{o}$-curve intersects its average AD curve at the same time (*t* = 2500) that the *τ*-curve intersects its own average AD curve; the intersection points are marked with blue diamonds. At this time, $\left(A_{\beta }^{o},\;\tau \right)=\left(6.70\times 10^{-6},\;4.90\times 10^{-10}\right)$, and we assume that the patient is then in an advanced state of MCI. We also assume that the untreated person would have been diagnosed as AD patient by t=3600d, where $\left(A_{\beta }^{o},\;\tau \right)=\left(7.10\times 10^{-6},\;5.24\times 10^{-10}\right)$, or earlier.

To observe the effect of the drug on the densities of $A_{\beta }^{o}$ and *τ* when the treatment begins early, we consider two start times,

$$\begin{array}{cc} & \left(A_{\beta }^{o},\;\tau \right)=\left(6.42\times 10^{-6},\;4.66\times 10^{-10}\right)\;\;\text{at}\;\;t=2000\;d,\;\;\text{and} \end{array}$$ (17)

$$\begin{array}{c} \left(A_{\beta }^{o},\;\tau \right)=\left(6.60\times 10^{-6},\;4.81\times 10^{-10}\right)\;\;\text{at}\;\;t=2300\;d. \end{array}$$ (18)

Fig 10 shows that early treatment which begins at t=2300d delays the advanced state of MCI by 200–300 days, while with an earlier treatment, at t=2000d, the delay is about 500–600 days. Of course, with an earlier start of a treatment, there is more uncertainty that the patient is going to be diagnosed with AD some years later.

## Conclusion

Alzheimer’s disease (AD) is a complex neurodegenerative disorder characterized by progressive cognitive decline, memory loss, and impairment of daily functioning. Brain PET scans of AD patients show abnormal aggregations of plaques of amyloid-*β* (*Aβ*) and neurofibrillary tangles of hyperphosphorylated tau proteins (*τ*). While the cause of the disease is not known, there are several hypotheses about early or earlier events, most commonly the amyloid hypothesis and the tau hypothesis. Inflammation is also an early event. Most patients visit the hospital after the onset of clinical symptoms. However, the earlier pathological asymptotic indications of AD appear decades, before the onset of clinical symptoms [10]. During that period, the hallmarks of the disease are accumulation of *Aβ* and *τ*. Longitudinal studies try to understand the early dynamics of (*Aβ*,*τ*) that may lead to AD.

In this paper, we introduced a mathematical model that can produce simulated patterns of (*Aβ*,*τ*) seen in PET scans of AD patients. The model is based on the assumption that the early event of inflammation drives the dynamics of (*Aβ*,*τ*). We represent inflammation of *Aβ* and *τ*, respectively, in the form

$$\begin{array}{c} R=R\left(x,y\right)\frac{t/\gamma }{K+t},\;\;\text{and}\;\;\bar{R}=\bar{R}\left(x,y\right)\frac{t/\gamma }{K+t}, \end{array}$$

for some positive constants *K*, *γ*, where (*x*, *y*) is variable point in the brain. The model shows that the profile of early inflammation determines the future profiles of *Aβ* and *τ* and their relative densities. We also demonstrate that the model can produce patterns of (*Aβ*,*τ*) seen in PET scans of AD patients.

We used the model in longitudinal studies of AD that aim to explore the optimal time to start treatment of AD with drugs that slow the progression of the disease, before damage had already occurred to the brain. We showed how the model can be used to estimate the time delay in the progressive growth of (*Aβ*,*τ*), when treatment begins as soon as a patient shows early MCI symptoms, or earlier.

The mathematical model does not address the mechanisms of plaque formation of *Aβ* peptides, and the formation of neurofibrillary tangles of *τ*. But it could nevertheless be useful in the planning phase of future longitudinal clinical studies of AD.

## Supporting information

S1 File Model equations, parameter estimates, numerical method, and tables of parameters. (ZIP)
