# Supplementary material for: Generating PET scan patterns in Alzheimer’s by a mathematical model
Source: PLoS One. 2024 Apr 16;19(4):e0299637. doi: 10.1371/journal.pone.0299637 (PMC11020767; doi:10.1371/journal.pone.0299637)
Supplement: S1 File — (ZIP) [file pone.0299637.s001.zip › Alzheimer_plos_S1_Appendix_v6_revision_unmarked.pdf]

Generating PET scan patterns in Alzheimer's by a mathematical model: Supplementary Information

Chaeyoung Lee[1*], Avner Friedman[2]

1 Department of Mathematics, Kyonggi University, Suwon 16227, Republic of Korea
2 Department of Mathematics, The Ohio State University, Columbus, OH, USA

* Corresponding author.
Email: cylee@kyonggi.ac.kr (CL)

A Model

A.1 Equation for A_β^i

The vulnerability of the brain to ROS is a key factor and early event driving AD [1]. $A\beta$ is constitutively produced in neurons upon cleavage of membrane soluble amyloid precursor protein (sAPP) into smaller fragments (peptides) and A_β^i [2,3]. We write the equation for A_β^i in the following form:

$$\frac{\partial A_\beta^i}{\partial t} - D_{A_\beta^i}\nabla^2 A_\beta^i = \left[R + \lambda_{A_\beta^i} - d_{A_\beta^i} A_\beta^i\right]\frac{N}{N_0}, \tag{1}$$

where R increases the proliferation of A_β^i; when $R=0$, $\lambda_{A_\beta^i} - d_{A_\beta^i}{A_\beta^i}^{ss} = 0$, where ${A_\beta^i}^{ss}$ is the steady state of A_β^i in health.

A.2 Equation for A_β^o

A_β^o are the extraneural Aβ that form the plaque seen near neurons in AD patients. We write the equation for A_β^o in the following form:

$$\frac{\partial A_\beta^o}{\partial t} - D_{A_\beta^o}\nabla^2 A_\beta^o = \lambda_{A_\beta^o} - \lambda_{A_\beta^o N}\frac{1}{N}\frac{\partial N}{\partial t}A_\beta^i - d_{A_\beta^o M} M A_\beta^o - d_{A_\beta^o} A_\beta^o + \lambda_{A_\beta^o A} A A_\beta^o, \tag{2}$$

where $\partial N/\partial t$ is the death rate ($-\partial N/\partial t > 0$).

A.3 Equation for τ and F_i

Tau proteins are constitutively expressed in neurons [4]. Excessive A_β^i activates GSK-3 through dephosphorylation, and activated GSK-3 promotes hyperphosphorylation of tau [5,6], which results in formation of NFT [7,8]. We can write the equation for tau as follows:

$$\frac{\partial \tau}{\partial t} - D_\tau\nabla^2\tau = \left[\bar{R} + \lambda_\tau + \lambda_{\tau A_\beta^i}(A_\beta^i - {A_\beta^i}^{ss})^+ - d_\tau \tau\right]\frac{N}{N_0}, \tag{3}$$

where $\bar{R}$ increases the proliferataion of τ, and we use the notation: $X^+ = X$ if $X \geq 0$, $X^+ = 0$ if $X < 0$. Note that if $A_\beta^i = {A_\beta^i}^{ss}$, then $\lambda_\tau - d_\tau \tau^{ss} = 0$, where τ^{ss} is the steady state of tau protein in health.

We assume, as in [9], that 60% of hyperphosphorylated tau proteins are involved in the formation of the neurofibrillary tangles, so that

$$F_i(t) = 0.6(\tau - \tau^{ss}). \tag{4}$$

A.4 Equation for M

Microglia cells are highly dynamic both in normal and pathological brain conditions [10]. Microglia mobility in AD is directed toward a source of injury [11], which we take to be the accumulation of A^o_β. Microglia are activated by A^o_β [12] and NFT [13]. Hence,

$$\frac{\partial M}{\partial t} + \delta_{MA^o_\beta} \nabla \cdot \left(M \frac{\nabla A^o_\beta}{K_{\nabla A^o_\beta} + |\nabla A^o_\beta|} \right) - D_M \nabla^2 M$$
$$= \lambda_M + M \left(\lambda_{MA^o_\beta} \frac{(A^o_\beta - A^{o\,ss}_\beta)^+}{K_{A^o_\beta} + (A^o_\beta - A^{o\,ss}_\beta)^+} + \lambda_{MF_i} \frac{F_i}{K_{F_i} + F_i} \right) - d_M M. \quad (5)$$

A.5 Equation for A

Astrocytes are glial cells that support neurons in homeostasis. In AD, they are activated by inflammatory cytokines secreted by microglia, and activated astrocytes stimulate Aβ formation by cleaving their APP [14]. Other functions of subpopulations of activated astrocytes have been studied [14–16], but it is not clear, at this time, what is their total effect on AD progression. We can then write the equation for A in the form:

$$\frac{dA}{dt} = \lambda_A + \lambda_{AM} \frac{M}{K_M + M} A - d_A A. \quad (6)$$

A.6 Equation for N

NFT causes death of neurons [17]. It is widely thought that amyloid plaques also contribute to the death of neurons in people with Alzheimer's [18]. In particular, $A\beta$ 42/40 are detected in Alzheimer's amyloid plaques [19], and $A\beta$-42 induces apoptosis in neurons by targeting their mitochondria [20]. Furthermore, A^o_β causes inflammation indirectly, for example, by activating microglia, who produce inflammatory cytokines [21,22], which further damages neurons. We write the equation for N in the following form:

$$\frac{\partial N}{\partial t} = -d_{NF_i} \frac{F_i}{K_{F_i} + F_i} N - d_{NA^o_\beta} \frac{(A^o_\beta - A^{o\,ss}_\beta)^+}{K_{A^o_\beta} + (A^o_\beta - A^{o\,ss}_\beta)^+} N, \quad N(0) = N_0. \quad (7)$$

A.7 Clinical data

The most common human brain cells are neurons and glial cells. There are approximately 100 billion neurons in adult humans, and at least as many glial cells [23]. Taking the mass one neuron to be 10^{-9} g, and noting that the brain volume is 1,500 cm^3, we find that the density of neurons in health is

$$N_0 = 6.00 \times 10^{-2} \text{ g/cm}^3.$$

Microglia make 6% of all brain cells [24], which we take to be 200 billion, and hence their density is approximately 12% of the density of neurons, so that

$$M_0 = 7.20 \times 10^{-3} \text{ g/cm}^3.$$

Astrocytes are four times as many as microglia [25]. Hence,

$$A_0 = 2.88 \times 10^{-2} \text{ g/cm}^3.$$

The number of neurons decreases by approximately 34% over the entire period of AD [26]. However, life expectancy at diagnosis varies greatly [27–29]. We take it to be in the range of 5-20 years, with an average of 10 years [27], so that

$$N(t) = 3.96 \times 10^{-2} \text{ g/cm}^3 \text{ at } t = 10 \text{ years}. \quad (8)$$

Assuming that this corresponds to the constant death rate d_N, then, for life expectancy of 15 years, the death rate of N will be $\frac{10}{20}d_N$, resulting in $N(t) = 4.87 \times 10^{-2}$ at $t = 10$ years; and for life expectancy of 5 years, death rate will be $\frac{10}{5}d_N$, so that $N(t) = 2.61 \times 10^{-2}$ at $t = 10$ years. (Of course, the simulation in this case will stop after 5 years.) Hence,

$$\text{Average } N(t) = 3.96 \times 10^{-2} \text{ g/cm}^3 \ \ (\text{range } 2.61 \times 10^{-2} - 4.87 \times 10^{-2}) \text{ in AD.} \tag{9}$$

In AD, microglia show high proliferation and differentiation [30, 31]; we take the range of microglia density to be, approximately, 1.5-3 times the density in homeostasis. The concentration of the pro-inflammatory monocytes is approximately 2.2 larger in AD than in health [32]. Accordingly, we take

$$\text{Average } M = 15.84 \times 10^{-3} \text{ g/cm}^3 \ \ (\text{range } 9.8 \times 10^{-3} - 19.8 \times 10^{-3}) \text{ in AD.} \tag{10}$$

Concentration of Aβ in gray matter was reported in [33], in health and in AD, as follows:

$$\begin{aligned} A_\beta^o &= 1,000 \text{ ng/g } (50 - 3,500 \text{ range}) \ \text{ in health,} \\ A_\beta^o &= 6,700 \text{ ng/g } (1,100 - 23,000 \text{ range}) \ \text{ in AD.} \end{aligned} \tag{11}$$

Concentration of tau protein was reported in [34], in health and in AD:

$$\begin{aligned} \tau &= 137 \text{ pg/ml } (50 - 300 \text{ range}) \ \text{ in health,} \\ \tau &= 490 \text{ pg/ml } (300 - 1,000 \text{ range}) \ \text{ in AD.} \end{aligned} \tag{12}$$

A.8 Inflammation formulae

We assume that inflammation begins at day 0 and increases with time, taking

$$R(t) = R\frac{t}{100 + t}, \quad \text{and} \quad \bar{R}(t) = \bar{R}\frac{t}{100 + t}; \tag{13}$$

in health both R and $\bar{R}$ are equal to 0.

A.9 Boundary condition

We next proceed with PDE simulations in a two-dimensional domain Ω using the no-flux boundary conditions,

$$\frac{\partial A_\beta^i}{\partial \mathrm{n}} = 0, \quad \frac{\partial A_\beta^o}{\partial \mathrm{n}} = 0, \quad \frac{\partial \tau}{\partial \mathrm{n}} = 0, \quad \frac{\partial M}{\partial \mathrm{n}} = 0, \tag{14}$$

where n is the outer normal vector at the boundary $\partial\Omega$.

B Parameter estimates

The diffusion coefficient of A_β^o in water is in the range of $(1.4 \times 10^{-6} - 2.1 \times 10^{-6})$ cm^2/s $\sim$ 1.2×10^{-1} cm^2/d [35], but should be much smaller for A_β^o aggregates, and for A_β^i in neurons. Surface diffusion of A_β monomer is 7.8×10^{-8} cm^2/s $\sim 6.2 \times 10^{-3}$ cm^2/d [36]; we assume that diffusion of A_β^o is smaller, and take

$$D_{A_\beta^i} = D_{A_\beta^o} = 7.85 \times 10^{-4} \text{ cm}^2/\text{d}.$$

The diffusion coefficient of free τ in neuron is approximately 3 μm^2/s $\sim 4 \times 10^{-3}$ cm^2/d [37], but smaller for τ on microtubules; we take,

$$D_\tau = 1.65 \times 10^{-3} \text{ cm}^2/\text{d}.$$

Microglias, in adults, undergo small diffusion [38, 39]; we take

$$D_M = 4.00 \times 10^{-7}\ \mathrm{cm}^2/\mathrm{d}.$$

We take the chemotaxis force of A^o_β on M to be

$$\delta_{MA^o_\beta} = 3.00 \times 10^{-3}\ \mathrm{cm}/\mathrm{d}.$$

The degradation rate (or death rate) of species X is related to its half-life $t_{1/2}(X)$ by the formula

$$d_X = \frac{\ln 2}{t_{1/2}(X)}.$$

Half-life of $A\beta$ $\big(t_{1/2}(A\beta)\big)$ is 9 hours [40]. Hence

$$d_{A^i_\beta} = d_{A^o_\beta} = \frac{\ln 2}{(9/24)}\ \mathrm{d}^{-1} = 1.85\ \mathrm{d}^{-1}.$$

Half-life of tau protein in humans is 5-60 hours [41], and the average half-life in mice is 12 hours ([42] Table 1). We take

$$d_\tau = \frac{\ln 2}{0.5}\ \mathrm{d}^{-1} = 1.39\ \mathrm{d}^{-1}.$$

Half-life of inflammatory macrophages is 3 weeks [43], while microglia's half-life is much larger, since their renewal rate is slow, 28% per year [44]. Since, in AD, peripheral macrophages are known to migrate into the brain [45, 46], we account for this difference by taking $t_{1/2}(M) = 6$ weeks. Hence,

$$d_M = \frac{\ln 2}{42} = 1.65 \times 10^{-2}\ \mathrm{d}^{-1}.$$

The half-life of astrocytes is 161 d [47]. Hence,

$$d_A = \frac{\ln 2}{161} = 4.31 \times 10^{-3}\ \mathrm{d}^{-1}.$$

We denote the steady state in health ($R = \bar{R} = 0$) of A^i_β, A^o_β and τ, by ${A^i_\beta}^{ss}$, ${A^o_\beta}^{ss}$ and τ^{ss}, respectively. We assume that, in cleavage of APP in health, ${A^i_\beta}^{ss} = {A^o_\beta}^{ss}$. Assuming average tissue density of 1 g/cm^3, we find from Eqs. (11)–(12), in health, that

$$\begin{aligned} {A^o_\beta}^{ss} &= 1.00 \times 10^{-6}\ \mathrm{g/cm}^3, \\ {A^i_\beta}^{ss} &= 1.00 \times 10^{-6}\ \mathrm{g/cm}^3, \quad \text{and} \\ \tau^{ss} &= 1.37 \times 10^{-10}\ \mathrm{g/cm}^3. \end{aligned}$$

In health, $R = 0$, so that $\lambda_{A^i_\beta} - d_{A^i_\beta}{A^i_\beta}^{ss} = 0$; hence,

$$\lambda_{A^i_\beta} = d_{A^i_\beta}{A^i_\beta}^{ss} = (1.85\ \mathrm{d}^{-1}) \times (1.00 \times 10^{-6}\ \mathrm{g/cm}^3) = 1.85 \times 10^{-6}\ \mathrm{g}/(\mathrm{cm}^3 \cdot \mathrm{d}).$$

Similarly, in health, $\bar{R} = 0$ so that

$$\lambda_\tau = d_\tau \tau^{ss} = (1.39\ \mathrm{d}^{-1}) \times (1.37 \times 10^{-10}\ \mathrm{g/cm}^3) = 1.90 \times 10^{-10}\ \mathrm{g}/(\mathrm{cm}^3 \cdot \mathrm{d}).$$

In order to determine the order of magnitude of R, we note that for large t we get, from Eq. (1),

$$A^i_\beta(t) \sim \frac{\lambda_{A^i_\beta} + R}{d_{A^i_\beta}} = {A^i_\beta}^{ss} + \frac{R}{d_{A^i_\beta}}.$$

Similarly, from Eq. (2) we get, for large t,

$$\tau(t) \sim \left(\bar{R} + \lambda_\tau + \frac{\lambda_{\tau A_\beta^i} R}{d_{A_\beta^i}}\right) / d_\tau = \frac{\bar{R}}{d_\tau} + \tau^{ss} + \frac{\lambda_{\tau A_\beta^i} R}{d_{A_\beta^i} d_\tau}. \tag{15}$$

We take

$$\lambda_{\tau A_\beta^i} = 7.76 \times 10^{-5}\ \mathrm{d}^{-1},$$

and then choose $R = 1.85 \times 10^{-6}$ g/(cm$^3 \cdot$ d) and $\bar{R} = 4.13 \times 10^{-10}$ g/(cm$^3 \cdot$ d), in order to get the asymptotic value of $\tau(t)$ to agree with the average of τ in AD.

We take

$$\begin{aligned} \lambda_{MA_\beta^o} &= 8.85 \times 10^{-3}\ \mathrm{d}^{-1}, \quad \text{and} \\ \lambda_{MF_i} &= 8.75 \times 10^{-3}\ \mathrm{d}^{-1}, \\ K_M &= 1.44 \times 10^{-2}\ \mathrm{g/cm}^3, \end{aligned}$$

in order to fit the increase of microglia in AD to the clinical data in Eq. (10).

We take the activation rate of astrocytes, by microglia-induced cytokines, to be

$$\lambda_{AM} = 4.28 \times 10^{-3}\ \mathrm{d}^{-1}.$$

The rate coefficients in growth and degradation of A_β^o are unknown; we take

$$\begin{aligned} \lambda_{A_\beta^o N} &= 6.19 \times 10^4, \\ \lambda_{A_\beta^o A} &= 2.50 \times 10^1\ (\mathrm{g/cm}^3)^2 \cdot \mathrm{d}^{-1}, \\ d_{A_\beta^o M} &= 9.90 \times 10^1\ (\mathrm{g/cm}^3)^2 \cdot \mathrm{d}^{-1}, \end{aligned}$$

and, with this choice, we are able to derive the steady state of A_β^o to agree with the clinical data in Eq. (11).

We take

$$\begin{aligned} K_{\nabla A_\beta^o} &= 1.00 \times 10^{-4}\ \mathrm{cm}^2/\mathrm{d}, \\ K_{A_\beta^o} &= 5.50 \times 10^{-6}\ \mathrm{g/cm}^3, \\ K_{F_i} &= 2.00 \times 10^{-10}\ \mathrm{g/cm}^3, \end{aligned}$$

and assume that in steady state $\frac{F_i}{K_{F_i}+F_i} \sim \frac{1}{2}$, $\frac{(A_\beta^o - A_\beta^{o\,ss})}{K_{A_\beta^o} + (A_\beta^o - A_\beta^{o\,ss})} \sim \frac{1}{2}$; this turns out to be consistent with the ODE simulation results in Fig. 2. Taking also

$$d_{NF_i} = d_{NA_\beta^o} = d_N,$$

Eq. (7) becomes

$$\frac{dN}{dt} = -d_N N.$$

Taking $d_N = 1.14 \times 10^{-4}\ \mathrm{d}^{-1}$, we get

$$N(3650) = N_0 e^{-3650 d_N} = 0.66 N_0 = 3.96 \times 10^{-2},$$

in close agreement with the clinical data in Eq. (9).

From Eqs. (2), (5), and (6) in steady state in health, we get

$$\lambda_{A_\beta^o} - (d_{A_\beta^o M} M_0 + d_{A_\beta^o} - \lambda_{A_\beta^o A} A_0) {A_\beta^o}^{ss} = 0,$$
$$\lambda_M - d_M M_0 = 0,$$
$$\lambda_A + \lambda_{AM} \frac{M_0}{K_M + M_0} A_0 - d_A A_0 = 0.$$

Hence,

$$\lambda_{A_\beta^o} = 1.84 \times 10^{-6} \text{ g/(cm}^3 \cdot \text{d)},$$
$$\lambda_M = 1.19 \times 10^{-4} \text{ g/(cm}^3 \cdot \text{d)},$$
$$\lambda_A = 8.30 \times 10^{-5} \text{ g/(cm}^3 \cdot \text{d)}.$$

C Numerical method

In this section, we describe discretization and an explicit Euler method for numerically solving the governing system (1)–(7) with the boundary conditions (14) on a two-dimensional domain $\Omega = (a, b) \times (c, d)$. The computational domain is partitioned into a uniform mesh with mesh spacing h, and the discrete domain Ω_h is defined as $\Omega_h = \{(x_p, y_q) \mid x_p = a + (p - 0.5)h,\ y_q = c + (q - 0.5)h,\ \text{for } 1 \leq p \leq N_x,\ 1 \leq q \leq N_y,\ N_x \in \mathbb{Z},\ N_y \in \mathbb{Z}\}$, which is a set of cell-center points. Let u_{pq}^k be an approximation of $u(x_p, y_q, k\Delta t)$, where $\Delta t = T/N_t$ is the time step size, T is the final time, and N_t is the number of time steps. In this paper, we use $h = 10^{-2}$ and $\Delta t = 10^{-4}$.

We define the discrete differentiation, gradient, divergence, and Laplace operators, for $1 \leq p \leq N_x$ and $1 \leq q \leq N_y$, as follows:

$$D_x u_{p+\frac{1}{2},q} = \frac{u_{p+1,q} - u_{pq}}{h}, \quad D_y u_{p,q+\frac{1}{2}} = \frac{u_{p,q+1} - u_{pq}}{h},$$
$$\nabla_h u_{pq} = \left(\frac{u_{p+\frac{1}{2},q} - u_{p-\frac{1}{2},q}}{h}, \frac{u_{p,q+\frac{1}{2}} - u_{p,q-\frac{1}{2}}}{h} \right),$$
$$\nabla_h \cdot (u, v)_{pq} = \frac{D_x u_{p+\frac{1}{2},q} - D_x u_{p-\frac{1}{2},q}}{h} + \frac{D_y v_{p,q+\frac{1}{2}} - D_y v_{p,q-\frac{1}{2}}}{h},$$
$$\nabla_h^2 u_{pq} = \nabla_h \cdot \nabla_h u_{pq}.$$

Here, we use the no-flux boundary condition, so that

$$u_{0,q} = u_{1,q},\ u_{N_x+1,q} = u_{N_x,q},\ \text{for } 1 \leq q \leq N_y,$$
$$u_{p,0} = u_{p,1},\ u_{p,N_y+1} = u_{p,N_y},\ \text{for } 0 \leq p \leq N_x + 1.$$

The initial conditions is taken as ${A_\beta^i}^0 = {A_\beta^i}^{ss}$, ${A_\beta^o}^0 = {A_\beta^o}^{ss}$, $\tau^0 = \tau^{ss}$, ${F_i}^0 = 0$, $M^0 = M_0$, $A^0 = A_0$, and $N^0 = N^{-1} = N_0$ for $1 \leq p \leq N_x$ and $1 \leq q \leq N_y$. We compute the following discrete system for

$0 \le k \le N_t$ in the following order, using C language, and depict the figures using MATLAB R2021b:

$$\frac{{A^i_\beta}_{pq}^{k+1} - {A^i_\beta}_{pq}^{k}}{\Delta t} - D_{A^i_\beta}\nabla_h^2 {A^i_\beta}_{pq}^{k} = R^k_{pq} + \lambda_{A^i_\beta} - d_{A^i_\beta}{A^i_\beta}_{pq}^{k} + \lambda_{A^o_\beta N}\frac{1}{N^k_{pq}}\frac{N^k_{pq} - N^{k-1}_{pq}}{\Delta t}{A^i_\beta}_{pq}^{k}, \tag{16}$$

$$\begin{aligned}&\frac{{A^o_\beta}_{pq}^{k+1} - {A^o_\beta}_{pq}^{k}}{\Delta t} - D_{A^o_\beta}\nabla_h^2 {A^o_\beta}_{pq}^{k}\\ &\quad = \lambda_{A^o_\beta} - \lambda_{A^o_\beta N}\frac{1}{N^k_{pq}}\frac{N^k_{pq} - N^{k-1}_{pq}}{\Delta t}{A^i_\beta}_{pq}^{k} - d_{A^o_\beta M}M^k_{pq}{A^o_\beta}_{pq}^{k} - d_{A^o_\beta}{A^o_\beta}_{pq}^{k} + \lambda_{A^o_\beta A}A^k_{pq}{A^o_\beta}_{pq}^{k},\end{aligned} \tag{17}$$

$$\frac{\tau^{k+1}_{pq} - \tau^k_{pq}}{\Delta t} - D_\tau\nabla_h^2\tau^k_{pq} = \bar{R}^k_{pq} + \lambda_\tau + \lambda_{\tau A^i_\beta}({A^i_\beta}_{pq}^{k} - {A^i_\beta}^{ss})^+ - d_\tau\tau^k_{pq}, \tag{18}$$

$$F_{i_{pq}}^{k+1} = 0.6(\tau^{k+1}_{pq} - \tau^{ss}), \tag{19}$$

$$\begin{aligned}&\frac{\partial M}{\Delta t} + \delta_{MA^o_\beta}\nabla_h\cdot\left(M\frac{\nabla_h A^o_\beta}{K_{\nabla A^o_\beta} + |\nabla_h A^o_\beta|}\right)^k_{pq} - D_M\nabla_h^2 M^k_{pq}\\ &\quad = \lambda_M + M^k_{pq}\left(\lambda_{MA^o_\beta}\frac{({A^o_\beta}_{pq}^{k} - {A^o_\beta}^{ss})^+}{K_{A^o_\beta} + ({A^o_\beta}_{pq}^{k} - {A^o_\beta}^{ss})^+} + \lambda_{MF_i}\frac{F_{i_{pq}}^k}{K_{F_i} + F_{i_{pq}}^k}\right) - d_M M^k_{pq},\end{aligned} \tag{20}$$

$$\frac{A^{k+1}_{pq} - A^k_{pq}}{\Delta t} = \lambda_A + \lambda_{AM}\frac{M^k_{pq}}{K_M + M^k_{pq}}A^k_{pq} - d_A A^k_{pq}, \tag{21}$$

$$\frac{\partial N}{\Delta t} = -d_{NF_i}\frac{F_{i_{pq}}^k}{K_{F_i} + F_{i_{pq}}^k}N^k_{pq} - d_{NA^o_\beta}\frac{({A^o_\beta}_{pq}^{k} - {A^o_\beta}^{ss})^+}{K_{A^o_\beta} + ({A^o_\beta}_{pq}^{k} - {A^o_\beta}^{ss})^+}N^k_{pq}. \tag{22}$$

Here, $u_{p+\frac{1}{2},q} = 0.5(u_{p+1,q} + u_{pq})$ and $|\nabla_h u^k_{pq}| = ||\nabla_h u^k_{pq}||_2$ is $\mathcal{L}_2$-norm.

D Parameter summary

Table S1. Parameters for the model.

Parameters	Descriptions	Value	References
$D_{A^i_\beta}$	diffusion coefficient of A^i_β	7.85×10^{-4} cm^2/d	[36] est.
$D_{A^o_\beta}$	diffusion coefficient of A^o_β	7.85×10^{-4} cm^2/d	[36] est.
D_τ	diffusion coefficient of τ	1.65×10^{-3} cm^2/d	[37] est.
D_M	diffusion coefficient of M	4.00×10^{-7} cm^2/d	[38, 39] est.
$d_{A^i_\beta}$	decay rate of A^i_β	1.85 d^{-1}	[40] est.
$d_{A^o_\beta}$	decay rate of A^o_β	1.85 d^{-1}	[40] est.
$d_{A^o_\beta M}$	decay rate of A^o_β by M	9.90×10^{1} (g/cm^3)$^2 \cdot$ d^{-1}	fitted.
d_τ	decay rate of τ	1.39 d^{-1}	[41, 42] est.
d_M	death rate of M	1.65×10^{-2} d^{-1}	[43, 44] est.
d_A	death rate of A	4.31×10^{-3} d^{-1}	[47] est.
d_N	death rate of N	1.14×10^{-4} d^{-1}	[26, 27] est.
d_{NF_i}	death rate of N by F_i	1.14×10^{-4} d^{-1}	fitted.
$d_{NA^o_\beta}$	death rate of N by A^o_β	1.14×10^{-4} d^{-1}	fitted.
$\lambda_{A^i_\beta}$	production rate of A^i_β	1.85×10^{-6} g/(cm$^3 \cdot$ d)	est.
$\lambda_{A^o_\beta}$	production rate of A^o_β	1.84×10^{-6} g/(cm$^3 \cdot$ d)	est.
$\lambda_{A^o_\beta N}$	production rate of A^o_β by N	6.19×10^{4}	fitted.
λ_τ	production rate of τ	1.90×10^{-10} g/(cm$^3 \cdot$ d)	est.
$\lambda_{\tau A^i_\beta}$	production rate of τ by A^i_β	7.76×10^{-5} d^{-1}	est.
λ_M	production rate of M	1.19×10^{-4} g/(cm$^3 \cdot$ d)	est.
$\lambda_{MA^o_\beta}$	production rate of M by A^o_β	8.85×10^{-3} d^{-1}	fitted.
λ_{MF_i}	production rate of M by F_i	8.75×10^{-3} d^{-1}	fitted.
$\lambda_{A^o_\beta A}$	production rate of A^o_β by A	2.50×10^{1} (g/cm^3)$^2 \cdot$ d^{-1}	fitted.
λ_A	production rate of A	8.30×10^{-5} g/(cm$^3 \cdot$ d)	est.
λ_{AM}	production rate of A by M	4.28×10^{-3} d^{-1}	est.
$\delta_{MA^o_\beta}$	Chemotaxis force for M by A^o_β	3.00×10^{-3} cm/d	est.
$K_{\nabla A^o_\beta}$	Chemoattractant parameter of M by A^o_β	1.00×10^{-4} cm^2/d	est.
K_{F_i}	Michaelis–Menten parameter for F_i	2.00×10^{-10} g/cm^3	est.
$K_{A^o_\beta}$	Michaelis–Menten parameter for A^o_β	5.50×10^{-6} g/cm^3	est.
K_M	Michaelis–Menten parameter for M	1.44×10^{-2} g/cm^3	est.
R^*	R value in average-severe AD	1.85×10^{-6} g/(cm$^3 \cdot$ d)	est.
$\bar{R}^*$	$\bar{R}$ value in average-severe AD	4.13×10^{-10} g/(cm$^3 \cdot$ d)	est.
N_0	density of N in healthy normal brain	6.00×10^{-2} g/cm^3	[23] est.
M_0	steady state of M in health	7.20×10^{-3} g/cm^3	[23, 24] est.
A_0	steady state of A in health	2.88×10^{-2} g/cm^3	[23–25] est.
${A^i_\beta}^{ss}$	steady state of A^i_β in health	1.00×10^{-6} g/cm^3	[33] est.
${A^o_\beta}^{ss}$	steady state of A^o_β in health	1.00×10^{-6} g/cm^3	[33]
τ^{ss}	steady state of τ in health	1.37×10^{-10} g/cm^3	[34]
${A^o_\beta}^{AD}$	average state of A^o_β in AD	6.70×10^{-6} g/cm^3	[33]
τ^{AD}	average state of τ in AD	4.90×10^{-10} g/cm^3	[34]

- est.= this parameter was estimated in Section B.
- X^{ss} is steady state of X in health.
- fitted, to conform to clinical data.

References

1. Bhatt S, Puli L, Patil CR. Role of reactive oxygen species in the progression of Alzheimer's disease. Drug Discov. Today. 2021;26(3):794–803.
2. Hampel H, Hardy J, Blennow K, Chen C, Perry G, Kim SH, et al. The amyloid-β pathway in Alzheimer's disease. Mol. Psychiatr. 2021;26(10):5481–5503.
3. APP gene, MedlinePlus. April 11, 2022. Available online: https://medlineplus.gov/download/genetics/gene/app.pdf (accessed on August 14, 2023).
4. Iqbal K, Liu F, Gong CX, Grundke-Iqbal I. Tau in Alzheimer disease and related tauopathies. Curr. Alzheimer Res. 2010;7(8):656–664.
5. Hernandez F, Lucas JJ, Avila J. GSK3 and tau: two convergence points in Alzheimer's disease. J. Alzheimer's Dis. 2013;33(s1):S141–S144.
6. Zhang H, Wei W, Zhao M, Ma L, Jiang X, Pei H, et al. Interaction between Aβ and tau in the pathogenesis of Alzheimer's disease. Int. J. Biol. Sci. 2021;17(9):2181.
7. Ponce-Lopez T, Liy-Salmeron G, Hong E, Meneses A. Lithium, phenserine, memantine and pioglitazone reverse memory deficit and restore phospho-GSK3β decreased in hippocampus in intracerebroventricular streptozotocin induced memory deficit model. Brain Res. 2011;1426:73–85.
8. Wang JZ, Grundke-Iqbal I, Iqbal K. Kinases and phosphatases and tau sites involved in Alzheimer neurofibrillary degeneration. European J. Neurosci. 2007;25(1):59–68.
9. Hao W, Friedman A. Mathematical model on Alzheimer's disease. BMC Syst. Biol. 2016;10(1):108.
10. Parkhurst CN, Gan WB. Microglia dynamics and function in the CNS. Curr. Opin. Neurobiol. 2010;20(5):595–600.
11. Franco-Bocanegra DK, George B, Lau LC, Holmes C, Nicoll JA, Boche D. Microglial motility in Alzheimer's disease and after Aβ42 immunotherapy: a human post-mortem study. Acta Neuropathol. Commun. 2019;7(1):174.
12. Solito E, Sastre M. Microglia function in Alzheimer's disease. Front. Pharmacol. 2012;3:14.
13. Španić E, Langer Horvat L, Hof PR, Šimić G. Role of microglial cells in Alzheimer's disease tau propagation. Front. Aging Neurosci. 2019;11:271.
14. Monterey MD, Wei H, Wu X, Wu JQ. The many faces of astrocytes in Alzheimer's disease. Front. Neurol. 2021;12:619626.
15. Frost GR, Jonas LA, Li YM. Friend, foe or both? Immune activity in Alzheimer's disease. Front. Aging Neurosci. 2019;11:337.
16. Kumar A, Fontana IC, Nordberg A. Reactive astrogliosis: A friend or foe in the pathogenesis of Alzheimer's disease. J. Neurochem. 2023;164(3):309–324.
17. DeTure MA, Dickson DW. The neuropathological diagnosis of Alzheimer's disease. Mol. Neurodegener. 2019;14(1):32.
18. Wolvetang E. A step closer to understanding how brain cells die in Alzheimer's disease. Australian Institute for Bioengineering and Nanotechnology. 1 June 2018. Available online: https://aibn.uq.edu.au/article/2018/06/step-closer-understanding-how-brain-cells-die-alzheimers-disease (accessed on August 14, 2023).

19. Li Y, Schindler SE, Bollinger JG, Ovod V, Mawuenyega KG, Weiner MW, et al. Validation of plasma amyloid-β 42/40 for detecting Alzheimer disease amyloid plaques. Neurology. 2022;98(7):e688–e699.

20. Han XJ, Hu YY, Yang ZJ, Jiang LP, Shi SL, Li YR, et al. Amyloid β-42 induces neuronal apoptosis by targeting mitochondria. Mol. Med. Rep. 2017;16(4):4521–4528.

21. Morales I, Guzmán-Martínez L, Cerda-Troncoso C, Farías GA, Maccioni RB. Neuroinflammation in the pathogenesis of Alzheimer's disease. A rational framework for the search of novel therapeutic approaches. Front. Cell. Neurosci. 2014;8:112.

22. Chang R, Yee KL, Sumbria RK. Tumor necrosis factor α inhibition for Alzheimer's disease. J. Cent. Nerv. Syst. Dis. 2017;9:1–5.

23. Weaver EA II, Doyle H. Cells of the Brain. Dana Foundation, August 8, 2019. Available online: https://dana.org/article/cells-of-the-brain/ (accessible on August 14, 2023)

24. Dos Santos SE, Medeiros M, Porfirio J, Tavares W, Pessôa L, Grinberg L, et al. Similar microglial cell densities across brain structures and mammalian species: implications for brain tissue function. J. Neurosci. 2020;40(24):4622–4643.

25. Von Bartheld CS, Bahney J, Herculano-Houzel S. The search for true numbers of neurons and glial cells in the human brain: A review of 150 years of cell counting. J. Comp. Neurol. 2016;524(18):3865–3895.

26. Andrade-Moraes CH, Oliveira-Pinto AV, Castro-Fonseca E, da Silva CG, Guimarães DM, Szczupak D, et al. Cell number changes in Alzheimer's disease relate to dementia, not to plaques and tangles. Brain. 2013;136(12):3738–3752.

27. Alzheimer's society. The later stage of dementia. 18 June 2021, Available online: https://www.alzheimers.org.uk/about-dementia/symptoms-and-diagnosis/how-dementia-progresses/later-stages-dementia (Accessible on August 14, 2023)

28. Alzheimer's association. Alzheimer's stages - early, middle, late dementia symptoms. Available online: https://www.alz.org/alzheimers-dementia/stages (accessible on Ausust 14, 2023)

29. UT Southwestern Medical Center. UTSW study finds cognitive decline key factor in predicting life expectancy in Alzheimer's disease. March 14, 2022. Available online: https://www.utsouthwestern.edu/newsroom/articles/year-2022/cognitive-decline.html (accessible on August 14, 2023)

30. Hansen DV, Hanson JE, Sheng M. Microglia in Alzheimer's disease. J. Cell Biol. 2018;217(2):459–472.

31. Guan YH, Zhang LJ, Wang SY, Deng YD, Zhou HS, Chen DQ, Zhang LC. The role of microglia in Alzheimer's disease and progress of treatment. Ibrain. 2022;8(1):37–47.

32. Munawara U, Catanzaro M, Xu W, Tan C, Hirokawa K, Bosco N, et al. Hyperactivation of monocytes and macrophages in MCI patients contributes to the progression of Alzheimer's disease. Immun. Ageing. 2021;18(1):29.

33. Roher AE, Esh CL, Kokjohn TA, Castaño EM, Van GD, Vickle WM, et al. Aβ peptides in human plasma and tissues and their significance for Alzheimer's disease. Alzheimers Dement. 2009;5(1):18–29.

34. Kapaki E, Kilidireas K, Paraskevas GP, Michalopoulou M, Patsouris E. Highly increased CSF tau protein and decreased β-amyloid (1-42) in sporadic CJD: a discrimination from Alzheimer's disease? J. Neurol. Neurosurg. Psychiatry. 2001;71(3):401–403.

35. Waters J. The concentration of soluble extracellular amyloid-β protein in acute brain slices from CRND8 mice. PLoS One. 2010;5(12):e15709.

36. Cholko T, Barnum J, Chang CEA. Amyloid-β (Aβ42) peptide aggregation rate and mechanism on surfaces with widely varied properties: insights from brownian dynamics simulations. J. Phys. Chem. B. 2020;124(27):5549–5558.

37. Konzack S, Thies E, Marx A, Mandelkow EM, Mandelkow E. Swimming against the tide: mobility of the microtubule-associated protein tau in neurons. J. Neurosci. 2007;27(37):9916–9927.

38. Taquet M, Jankovski A, Rensonnet G, Jacobs D, Des Rieux A, Macq B, et al. Extra-axonal restricted diffusion as an in-vivo marker of reactive microglia. Sci. Rep. 2019;9(1):13874–13874.

39. Smolders SMT, Kessels S, Vangansewinkel T, Rigo JM, Legendre P, Brôône B. Microglia: Brain cells on the move. Prog. Neurobiol. 2019;178:101612.

40. Patterson BW, Elbert DL, Mawuenyega KG, Kasten T, Ovod V, Ma S, et al. Age and amyloid effects on human central nervous system amyloid-beta kinetics. Ann. Neurol. 2015;78(3):439–453.

41. Yamada K, Patel TK, Hochgräfe K, Mahan TE, Jiang H, Stewart FR, et al. Analysis of in vivo turnover of tau in a mouse model of tauopathy. Mol. Neurodegener. 2015;10(1):55.

42. Mandelkow EM, Mandelkow E. Biochemistry and cell biology of tau protein in neurofibrillary degeneration. Cold Spring Harb. Perspect. Med. 2012;2(7):a006247.

43. Italiani P, Boraschi D. From monocytes to M1/M2 macrophages: phenotypical vs. functional differentiation. Front. Immunol. 2014;5:514.

44. Réu P, Khosravi A, Bernard S, Mold JE, Salehpour M, Alkass K, et al. The lifespan and turnover of microglia in the human brain. Cell Reports 2017;20(4):779–784.

45. Wang Y, Subramanian M, Yurdagul A, Barbosa-Lorenzi VC, Cai B, de Juan-Sanz J, et al. Mitochondrial fission promotes the continued clearance of apoptotic cells by macrophages. Cell. 2017;171(2):331–345.

46. Augusto-Oliveira M, Arrifano GP, Lopes-Araújo A, Santos-Sacramento L, Takeda PY, Anthony DC, et al. What do microglia really do in healthy adult brain? Cells. 2019;8(10):1293.

47. Beccari S, Valero J, Maletic-Savatic M, Sierra A. A simulation model of neuroprogenitor proliferation dynamics predicts age-related loss of hippocampal neurogenesis but not astrogenesis. Sci. Rep. 2017;7(1):16528.
